# Diagnosis of Early Gastric Cancer by Magnifying Endoscopy with NBI from Viewpoint of Histological Imaging: Mucosal Patterning in terms of White Zone Visibility and Its Relationship to Histology

**DOI:** 10.1155/2012/954809

**Published:** 2012-12-03

**Authors:** Kazuyoshi Yagi, Yujiro Nozawa, Shinsaku Endou, Atsuo Nakamura

**Affiliations:** Department of Internal Medicine, Niigata Prefectural Yoshida Hospital, 959-0242 Niigata, Japan

## Abstract

The diagnosis of early gastric cancer by magnifying endoscopy with NBI is based on two components: microvascular pattern and mucosal pattern. Mucosal patterns are characterized by a whitish edge, which has been named the white zone. Some cancerous areas showing a distinct white zone form clear mucosal patterns, whereas others showing a nondistinct white zone do not form mucosal patterns. The aim of the present study was to clarify the histological differences between these two types of area. In transverse sections of gastric epithelium, the lengths of intervening parts in areas showing a distinct white zone, a nondistinct white zone, and an invisible white zone were measured, and the depths of the crypts in these three types of area were also measured. The intervening parts in areas with a nondistinct or invisible white zone were shorter than those in areas with a distinct white zone (*P* < 0.05), and the crypts in the former areas were shallower than those in the latter (*P* < 0.01). Areas in which the intervening part were long and the crypts deep tended to show a distinct white zone, whereas areas with short intervening parts or shallow crypts tended to show a nondistinct or non-visible white zone.

## 1. Introduction

**Figure 1:**
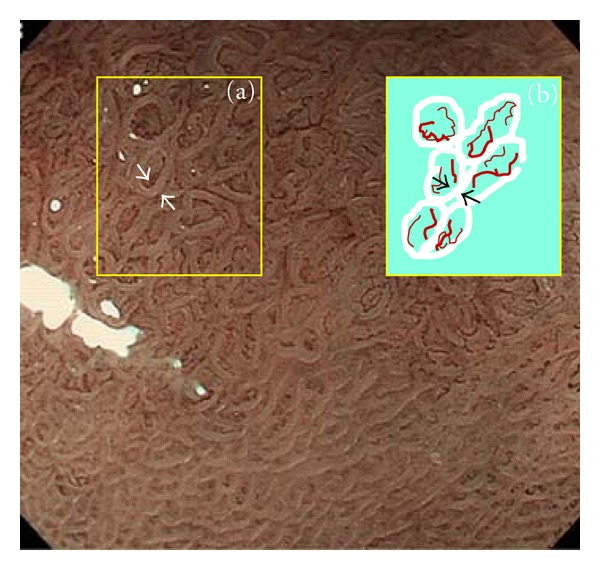
White zone inside yellow box (a) is indicated diagrammatically as white bold lines inside yellow box (b). Brownish lines indicate blood vessels. White zone is the area between the two white arrows in yellow box (a) and between the two black arrows in yellow box (b).

**Figure 2:**
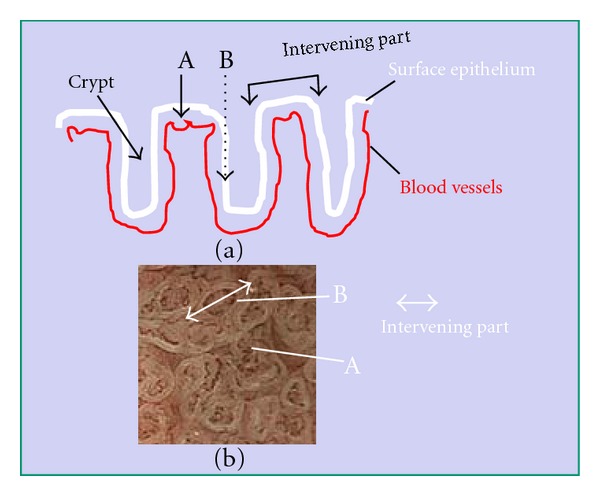
Mechanism responsible for visibility of the white zone when viewed perpendicularly above the epithelium. Black arrows A and B represent NBI light. As the mucosa is semi-translucent, A-NBI light passing through intervening part of the epithelium is absorbed by the hemoglobin of red blood cells in blood vessels. As a result, the blood vessels appear as brown lines, as seen in Figure 2(b)A. However, B-NBI light passing into the epithelia of crypts is scattered before it reaches any blood vessels, and thus this area appears as a white zone, as seen in Figure 2(b)B. Intervening part means the protruding area between crypts (a). In images obtained by magnifying endoscopy with NBI, this corresponds to space between two white zones forming the mucosal pattern, including the white zones themselves (b).

Magnifying endoscopy with narrowband imaging (NBI) has been used for diagnosis of gastric diseases because of its accuracy and applicability [1–4]. This technique is not only able to demonstrate microvascular patterns more clearly but also yields better images of mucosal patterns than magnifying endoscopy with white light. Mucosal patterning demonstrated by magnifying endoscopy with NBI provides considerable information about gastric lesions [3–7]. Our previous studies have shown that the mucosal pattern is characterized by a whitish edge (Figure 1), which we have named the “white zone” [8]. As mucosa is semitranslucent, NBI light passing through the intervening part between two crypts of epithelium is absorbed by the hemoglobin of red blood cells in blood vessels (Figure 2(a), arrow A). As a result, blood vessels appear as brown lines on the device monitor (Figure 2(b), A) [9]. However, as NBI light impinging on the epithelia of crypts is scattered before it reaches blood vessels (Figure 2(a), arrow B), a white zone is displayed (Figure 2(b), B) [9]. The intervening part is the protruding area between the crypts (Figure 2(a)). In images obtained by NBI-magnifying endoscopy, this corresponds to the space between two white zones forming a mucosal pattern and also the white zones themselves (Figure 2(b)). 

**Figure 3:**
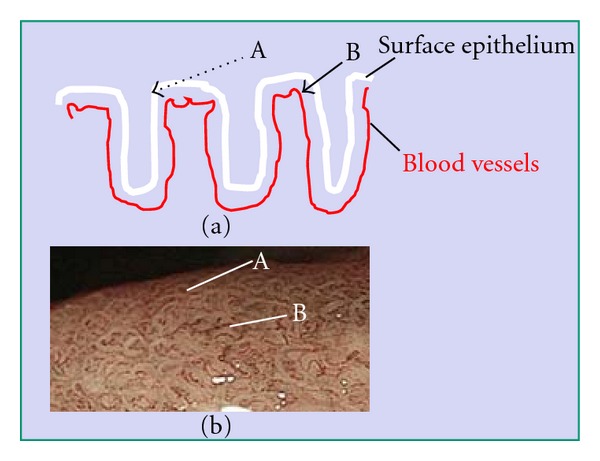
The mechanism responsible for white zone visibility at an angle. A-NBI light enters the mucosa from the intervening part in the direction of the crypt epithelium. It is then scattered, and the white zone appears as shown in Figure 3(b)A. B-NBI light entering crypts passes through the epithelium and is absorbed by the hemoglobin of red blood cells in blood vessels. As a result, the blood vessels appear as brown lines as shown in Figure 3(b)B.

On the other hand, if NBI light enters the mucosa at an angle, it is scattered between the intervening part and the crypt (Figure 3(a), arrow A) and this is demonstrated as a white zone (Figure 3(b), A) [9]. NBI light entering crypts passes through the epithelium and is absorbed by the hemoglobin of red blood cells in blood vessels (Figure 3(a), arrow B). As a result, blood vessels appear as brown lines (Figure 3(b), B) [9]. Therefore, we think that the white zone is observed under conditions when NBI-light passes through a layer of epithelium that is several cells thick. 

Magnifying endoscopy with NBI yields a sharply contrasted mucosal pattern in some gastric cancers because of the presence of a distinct white zone (Figure 1), whereas other gastric cancers show only a weakly contrasted pattern because the white zone is not distinct [6]. However, even in the latter case, a sharply contrasted mucosal pattern can be obtained after acetic acid has been sprayed onto the mucosa. Acetic acid changes the mucosa from semitranslucent to completely opaque, so that NBI light is reflected off the epithelial surface [10, 11]. This allows the structure of the mucosa to be observed in detail. Previously, we reported a study in which we compared the imaging obtained by NBI-magnifying endoscopy with that of conventional endoscopic imaging employing resected specimens that had been sprayed with acetic acid [6]. We found that cancerous glands in which magnifying endoscopy with NBI demonstrated a nondistinct white zone tended to have intervening parts of short length, indicating high gland density [6]. We also found that areas where glands had shallow crypts tend to show a nondistinct white zone. However, both types of areas showed a change to a sharply contrasted mucosal pattern after they had been sprayed with acetic acid [8]. 

**Figure 4:**
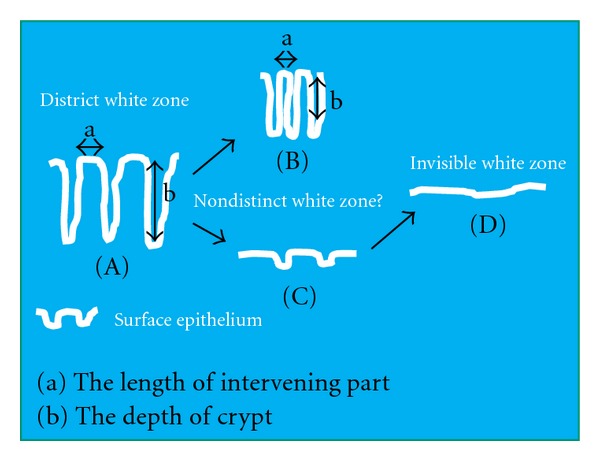
The relationship between white zone visibility, the length of intervening parts and crypt depth. In (A) the intervening part is long and the crypt is deep. In (B) the intervening part is short. In (C) the crypt is shallow with a long intervening part. In (D) no crypts are evident. Magnifying endoscopy with NBI would show a clear mucosal pattern with a distinct white zone in (A) and neither a mucosal pattern nor a white zone in (D) It is necessary to clarify how long the intervening part needs to be (B) and how deep the crypt needs to be (C) in order for the white zone to be distinct.

We considered that glands with a long intervening part and deep crypts (Figure 4(A)) show a distinct white zone by NBI-magnifying endoscopy, whereas flat epithelium without crypts (Figure 4(D)) fails to show a white zone. Furthermore, glands with a short intervening part (Figure 4(B)) and glands with shallow crypts (Figure 4(C)) are considered to show a nondistinct white zone and therefore do not show a sharply contrasted mucosal pattern. If it is possible to clarify the relationship between white zone visibility and surface epithelial structure in terms of the intervening part and crypts, it might become feasible to interpret the surface epithelial structure, thus representing an initial step towards histological-imaging diagnosis of lesions using magnifying endoscopy with NBI. 

In the present study, we investigated the length of the intervening part and the depth of crypts in areas showing a distinct white zone in comparison with that in areas showing no visible white zone or a nondistinct white zone. 

## 2. Materials and Methods

### 2.1. Relationship between White Zone Visibility and the Length of Intervening Parts

**Figure 5:**
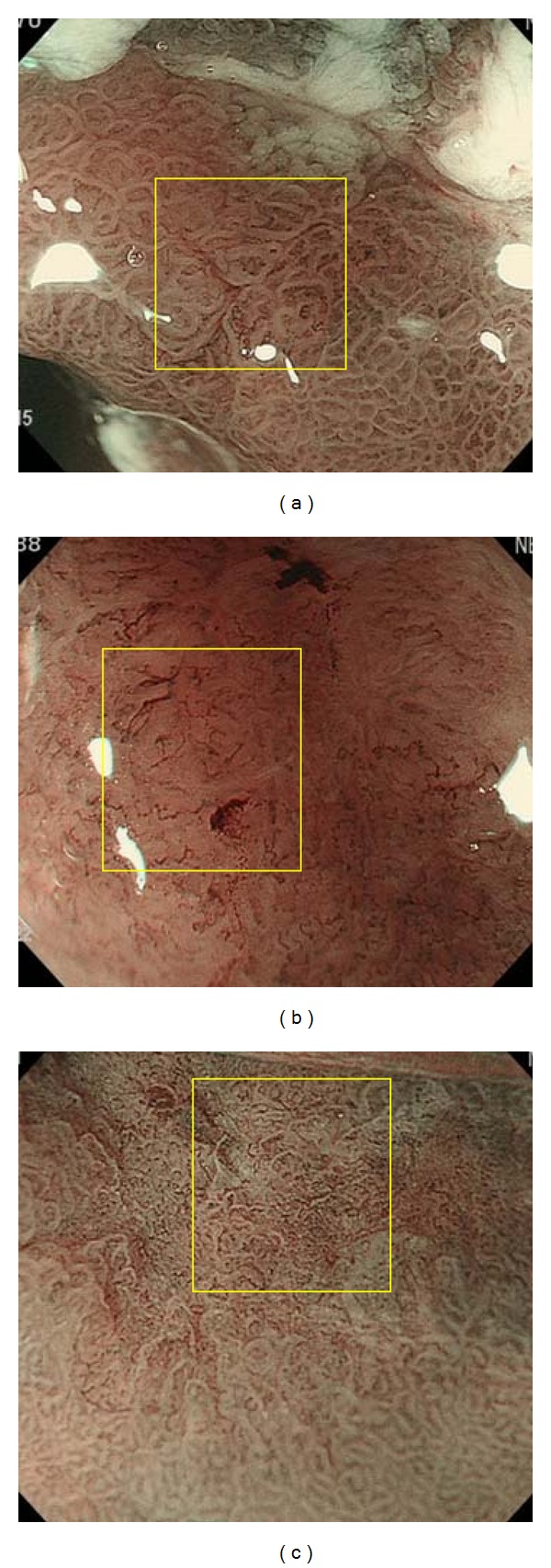
(a) A view obtained with NBI-magnifying endoscopy in which white zones are clearly observed (yellow box). (b) A view obtained with NBI-magnifying endoscopy in which white zones are not visible (yellow box). (c) A view obtained with NBI-magnifying endoscopy in which white zones are visible, but nondistinct (yellow box).

**Figure 6:**
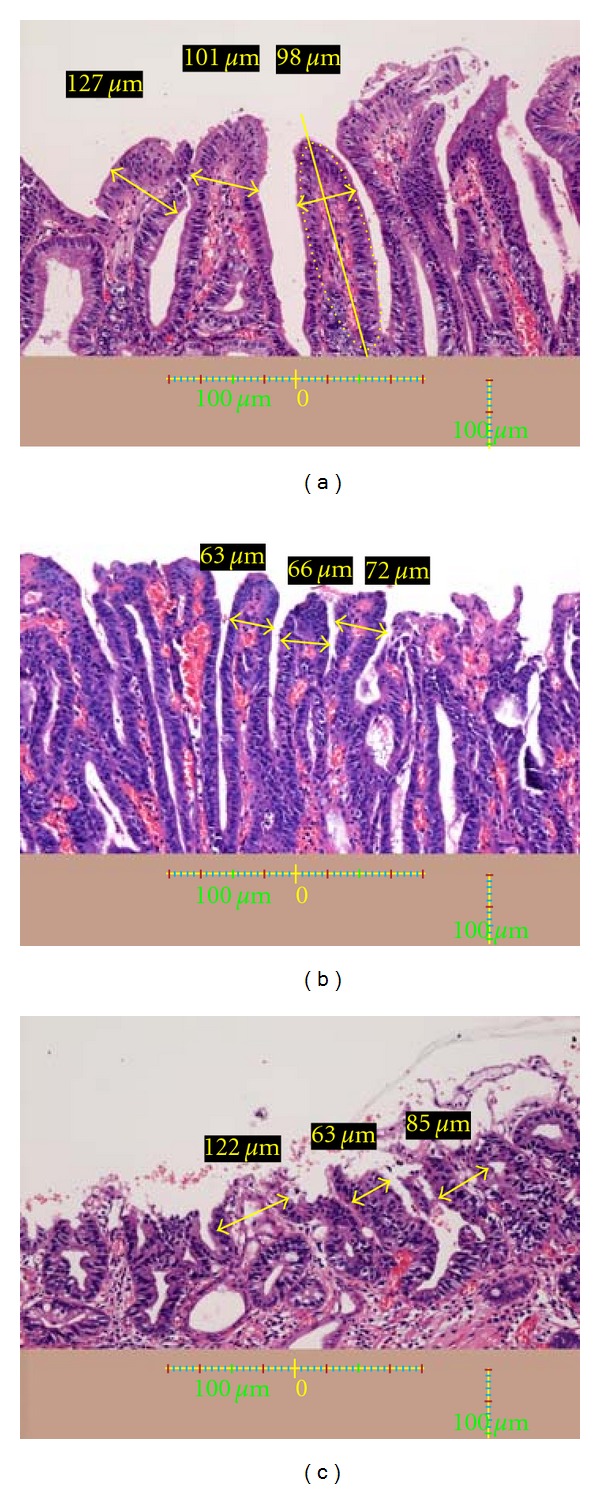
(a) A histological view of the yellow box in Figure 5(a). The lengths of three intervening parts are measured (yellow arrows and yellow figures). A scale bar is shown at the bottom. *Height and lengths of intervening parts*: The protruding parts between crypts have a shape resembling an ellipse (as indicated by the yellow dotted lined), allowing the major axis (yellow line) of the ellipse to be drawn. The vertical line representing the major axis was defined as the height of the intervening part. The width of the widest part less than 100 *μ*m from the top of the intervening parts was adopted as the length of the intervening parts. (b) A histological view of the yellow box in Figure 5(b). The lengths of three intervening parts are indicated (yellow arrows and yellow figures). A scale bar is shown at the bottom. (c) A histological view of the yellow box in Figure 5(c). The lengths of three intervening parts are indicated (yellow arrows and yellow figures). A scale bar is shown at the bottom.

Among gastric cancers that had been treated by endoscopic submucosal dissection (ESD) in our hospital, the following were selected on the basis of their appearance revealed by magnifying endoscopic imaging with NBI performed beforehand: 10 lesions showing a distinct white zone (Figure 5(a)), 10 lesions without any visible white zone (Figure 5(b)), and 10 lesions with a visible but nondistinct white zone (Figure 5(c)). In all cases, it had been confirmed that the histological areas examined corresponded to the parts observed by NBI-magnifying endoscopy. The resected specimens were examined using an Olympus BX50 microscope, and the lengths of the intervening parts of each type of cancerous mucosa in all 30 lesions were measured using a microscopic scale. Photographs of histological specimens were also taken using a Nikon DS L-1. The lengths of three intervening parts were measured in each lesion (Figures 6(a), 6(b), and 6(c)). Furthermore, the lengths of the intervening parts in the surrounding gastric mucosa, which exhibited gastritis, were also measured in all 30 lesions. White zones were clearly observed in all of the surrounding mucosal areas examined. 

The internal part of each intervening part between the crypts had a profile resembling an ellipse (Figure 6(a), area outlined by yellow dots), and this allowed the major axis (Figure 6(a), yellow line) of the ellipse to be drawn. The widest part vertical to the major axis less than 100 *μ*m from the top of the intervening parts was adopted as the length (Figures 6(a), 6(b), and 6(c), bold yellow line with arrow on both sides). Representative intervening parts showing the histological features of each type of area were chosen from one microscopic field beforehand and also as a rough measure.

### 2.2. Relationship between White Zone Visibility and Depth of Cancerous Crypts

**Figure 7:**
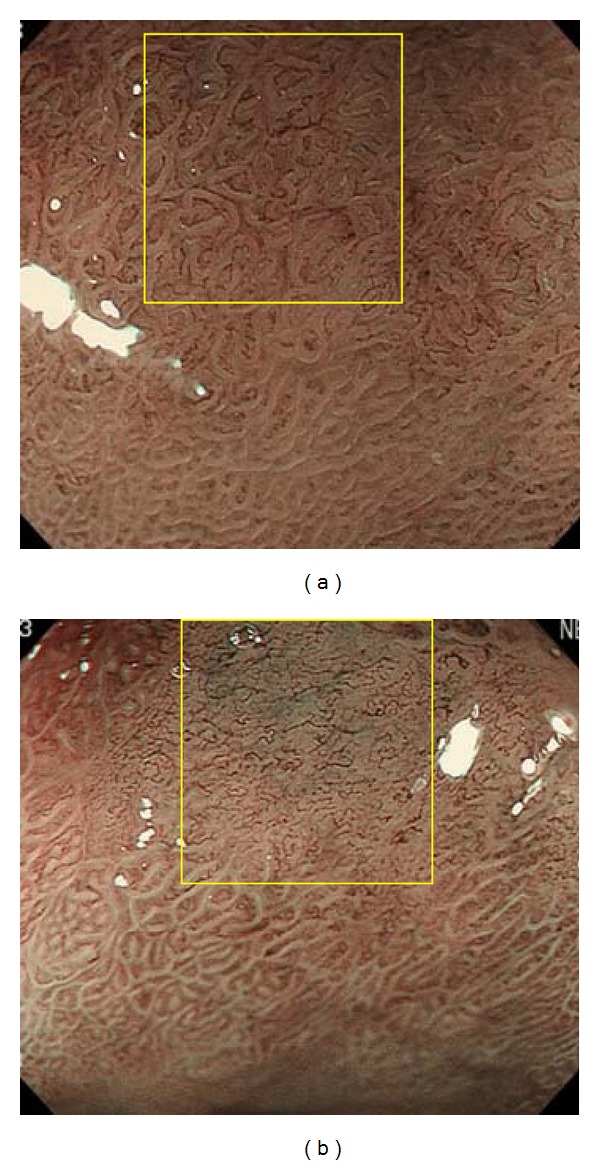
(a) A view obtained using NBI-magnifying endoscopy in which the white zone is clearly evident (yellow box). (b) A view obtained with NBI-magnifying endoscopy in which the white zone is not visible (yellow box).

Among gastric cancers that had been treated by ESD or surgery at our hospital, the following were selected on the basis of their appearance revealed by NBI-magnifying endoscopy beforehand: 12 lesions showing a distinct white zone (Figure 7(a)) and 12 other lesions in which the white zone was nondistinct or invisible (Figure 7(b)), the average width of the intervening parts being more than 100 *μ*m in both groups. When this second study was performed, we had already concluded that regions in which intervening parts were less than 100 *μ*m wide showed a nondistinct or invisible white zone. Since the aim of this second study was to ascertain whether lesions with shallow crypts showed a nondistinct or invisible white zone, we avoided lesions in which the intervening parts were less than 100 *μ*m wide because their white zone would have been nondistinct or invisible, regardless of crypt depth. 

As we were unable to obtain a sufficient number of photographs using NBI-magnifying endoscopy that were divisible into three groups based on the appearance of the white zone (distinct, nondistinct, or invisible), we investigated only two types of specimens in this second part of the study: those showing a distinct white zone and those showing a nondistinct or invisible white zone. 

**Figure 8:**
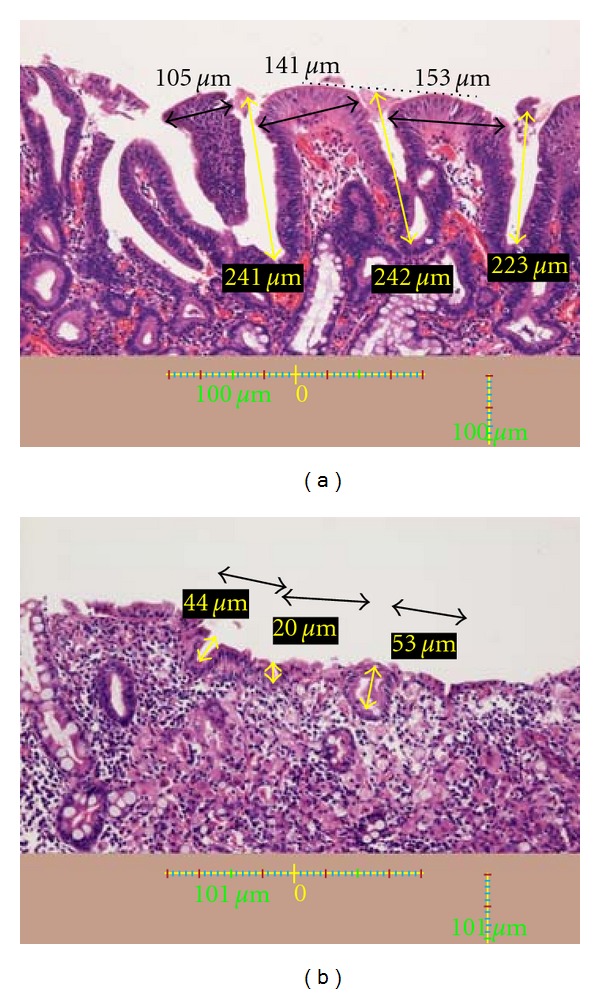
(a) A histological view of the yellow box in Figure 7(a). The depths of three crypts are indicated (yellow arrows and yellow figures). Black arrows and black figures indicate the lengths of the intervening parts. A scale bar is shown at the bottom. *Crypt*: On a line between the tops of two intervening parts (black dotted line), the point on the line over the center of a crypt was defined as the top of the crypt. The distance from this point to the bottom of the crypt was defined as the crypt depth. (b) A histological view of the yellow box in Figure 7(b). The depths of three crypts are indicated (yellow arrows and yellow figures). Black arrows and black figures indicate the lengths of the intervening parts. A scale bar is shown at the bottom.

The depths of three crypts in each lesion were measured using a Nikon DS L-1 (Figures 8(a) and 8(b)). Furthermore, the depths of crypts in the surrounding gastric mucosa, which showed evident gastritis, were measured for each of the 24 lesions. White zones were clearly observed in the surrounding mucosa in all cases. 

On a line joining the tops of two intervening parts (Figure 8(a), black dotted line), the point at the center of the line was defined as the top of the crypt. The depth of a crypt was defined as the distance from this point to the bottom of the crypt (Figures 8(a) and 8(b)). 

### 2.3. Relationship between White Zone Distinctiveness and Macroscopic Types of Gastric Cancer

The relationship between white zone distinctiveness and endoscopic macroscopic types was investigated. Macroscopic types of lesions were classified as elevated (type 0I or 0IIa), flat (type 0IIb), or depressive (type 0IIc) according to the Japanese Gastric Cancer Association classification of gastric cancer [12]. 

### 2.4. Statistical Analysis

Data are expressed as mean ± standard deviation. Differences in the widths of intervening parts and depths of crypts between groups were analyzed using Student's *t*-test. Differences at *P* < 0.05 were considered to be statistically significant. 

## 3. Results

### 3.1. Relationship between White Zone Distinctiveness and Length of Intervening Parts

The lengths of the intervening parts in lesions showing a distinct white zone, a visible but nondistinct white zone, or an invisible white zone were 136 ± 46 *μ*m, 74 ± 17 *μ*m, and 82 ± 34 *μ*m, respectively (Table 1). The length of the intervening parts in the surrounding gastric mucosa was 127 ± 46 *μ*m (Table 1). Intervening parts in lesions with a nondistinct white zone and those with an invisible white zone were significantly shorter than those in lesions with a distinctive white zone and also in the surrounding gastric mucosa (*P* < 0.05) (Table 1). 

### 3.2. Relationship between White Zone Distinctiveness and Depth of Cancerous Crypts

The depths of crypts in the glands of lesions showing a distinct white zone and those with a nondistinct or invisible white zone were 182 ± 52 *μ*m and 81 ± 25 *μ*m, respectively (Table 2). The depth of crypts in the surrounding gastric mucosa was 171 ± 42 *μ*m (Table 2). Crypts in lesions with a nondistinct or invisible white zone were significantly shallower than those in regions with a distinct white zone and in the surrounding gastric mucosa (*P* < 0.01) (Table 2).

### 3.3. Relationship between White Zone Distinctiveness and Macroscopic Types of Gastric Cancer

The number of lesions included in this study was 54, among which 22 showed a distinct white zone and 32 showed a nondistinct or invisible white zone. Among the lesions with a distinct white zone, 17 were elevated (type 0I or 0IIa), 2 were flat (type 0IIb), and 3 were depressed (type 0IIc). Among the lesions with a nondistinct or invisible white zone, 7 were elevated (type 0I or 0IIa), 3 were level (type 0IIb), and 22 were depressed (type 0IIc). 

## 4. Discussion

Nakayoshi et al. were the first to report the blood vessel patterns in gastric cancer visualized by magnifying endoscopy with NBI [13]. They referred to the mesh-like vascular appearance of differentiated adenocarcinoma as a “fine network pattern” and that in poorly differentiated adenocarcinoma as a “corkscrew pattern” [13]. Although that study demonstrated differences in blood vessel patterns according to the histological types of carcinoma, mucosal patterns observed using magnifying endoscopy with NBI received no attention at that time. 

A few years later, the mucosal pattern of gastric cancer demonstrated by magnifying endoscopy with NBI was reported as the “intrastructural irregular vessels” (ISIVs) pattern [14]. Other authors also reported an “intra-lobular loop” (ILL) [15] or “loop” [6] pattern in gastric cancer. 

Based on the use of NBI-magnifying endoscopy, our attention was drawn to the visibility of the mucosal pattern in some types of gastric cancer, and subsequently we studied the structure of the mucosa after spraying with acetic acid [6]. This revealed that lesions with a fine network pattern had densely arranged cylindrical glands with surrounding blood vessels forming a mesh-like pattern [6]. Therefore, we recognized that these blood vessels represented the mucosal pattern of cylindrical glands that is not visible by NBI-magnifying endoscopy. In addition, cancers in which the blood vessels formed a “dot and stick” pattern were found to have a villus-forming mucosal structure after spraying with acetic acid, even though the mucosal pattern was not visible [6]. On the other hand, lesions that showed a clear mucosal pattern exhibited no change in the pattern even after acetic acid spraying [6]. Thus it became apparent that there were two types of cancer in terms of the features demonstrated by NBI-magnifying endoscopy, one showing a clear mucosal pattern and the other an unclear or non-visible mucosal pattern. 

Through observation of many lesions using NBI-magnifying endoscopy, we noticed that those showing a clear mucosal pattern tended to have wide intervening parts and deep crypts, whereas those showing an unclear or invisible mucosal pattern tended to have short intervening parts, shallow crypts, or both. Since we hypothesized that it might be possible to grasp the outline of the surface epithelial structure of lesions by evaluating the visibility of the mucosal pattern, we named this outline the “white zone.”

Lesions in which the white zone is invisible or unclear have a short intervening part or shallow depth in comparison with lesions showing a clear white zone. As an example, lesions with a histology like that shown in Figure 4(b) or 4(c) are thought to have an unclear or invisible white zone, whereas those showing a histology like that shown in Figure 4(a) have a clear white zone. If magnifying endoscopy with NBI shows a clear mucosal pattern with a distinct white zone, then the surface epithelial structure has long intervening parts and deep crypts. 

Cancers have a tendency to possess dense glands, making the intervening parts naturally short. Furthermore, the crypts in cancerous glands are often shallow, because they tend to be immature and imperfectly formed. Therefore, in cancerous lesions, the white zone, which represents the mucosal structure, is often unclear or invisible by NBI-magnifying endoscopy. For the same reason, Kaise et al. have also reported that disappearance of the fine mucosal structure is one factor that can be used for specific diagnosis of superficial depressed gastric carcinoma [5]. In the present study, a nondistinct or invisible white zone tends to be demonstrated more frequently in depressive lesions than in elevated lesion. 

Thus, it is now possible to visualize the surface epithelial structure by magnifying endoscopic imaging with NBI, and the pattern of blood vessels can also yield more information about histology. This approach seems to have promise for histological examination based on the white zone and blood vessel patterning, for which we propose the term “histological-imaging diagnosis.”

In conclusion, recognition of the degree of visibility of the white zone is a first step towards histological-imaging diagnosis using magnifying endoscopy with NBI.

## Figures and Tables

**Table 1:** Relationship between white zone distinctiveness and length of the intervening part. The lengths of the intervening parts in areas where the white zones were distinct, visible but nondistinct, and invisible were 136 ± 46 *μ*m, 74 ± 17 *μ*m, and 82 ± 34 *μ*m, respectively. The length of the intervening parts in the surrounding gastritis mucosa was 127 ± 46 *μ*m. The lengths of the intervening parts of lesions showing a distinct white zone and those in the surrounding gastric mucosa were significantly longer than those in areas with a visible but nondistinct or a invisible white zone (*P* < 0.05).

Distinctness of white zone	The length of intervening parts
Lesion with distinct white zone	136 ± 46 *μ*m*
Lesions with visible, but nondistinct white zone	74 ± 17 *μ*m**
Lesion with invisible white zone	82 ± 34 *μ*m**
Surrounding gastric mucosa	127 ± 46 *μ*m*

*Versus **P < 0.05.

**Table 2:** Relationship between white zone distinctiveness and crypt depth. The depths of crypts in areas where the white zones were distinct and those in lesions where the white zones were nondistinct or invisible were 182 ± 52 *μ*m and 81 ± 25 *μ*m, respectively. The depth of crypts in the surrounding gastric mucosa was 171 ± 42 *μ*m. The crypts in lesions showing a distinct white zone and those in the surrounding gastric mucosa were significantly deeper than those in lesions with a nondistinct or invisible white zone (*P* < 0.01).

Distinctness of white zone	The depth of crypts
Lesion with distinct white zone	182 ± 52 *μ*m*
Lesions with nondistinct or invisible white zone	81 ± 25 *μ*m**
Surrounding gastric mucosa	171 ± 42 *μ*m*

*Versus **P < 0.01.

## References

[1] Ezoe Y, Muto M, Uedo N (2011). Magnifying narrow band imaging is more accurate than conventional white-light imaging in diagnosis of gastric mucosal cancer. *Gastroenterology*.

[2] Kato M, Kaise M, Yonezawa J (2010). Magnifying endoscopy with narrow-band imaging achieves superior accuracy in the differential diagnosis of superficial gastric lesions identified with white-light endoscopy: a prospective study. *Gastrointestinal Endoscopy*.

[3] Kawamura M, Abe S, Oikawa K (2011). Topographic differences in gastric micromucosal patterns observed by magnifying endoscopy with narrow band imaging. *Journal of Gastroenterology and Hepatology*.

[4] Nonaka K, Arai S, Ban S (2011). Prospective study of the evaluation of the usefulness of tumor typing by narrow band imaging for the differential diagnosis of gastric adenoma and well-differentiated adenocarcinoma. *Digestive Endoscopy*.

[5] Kaise M, Kato M, Urashima M (2009). Magnifying endoscopy combined with narrow-band imaging for differential diagnosis of superficial depressed gastric lesions. *Endoscopy*.

[6] Yagi K, Nakamura A, Sekine A, Umezu H (2008). Magnifying endoscopy with narrow band imaging for early differentiated gastric adenocarcinoma. *Digestive Endoscopy*.

[7] Kobayashi M, Takeuchi M, Ajioka Y (2011). Mucin phenotype and narrow-band imaging with magnifying endoscopy for differentiated-type mucosal gastric cancer. *Journal of Gastroenterology*.

[8] Yagi K, Sato T, Nakamura A (2009). The possibility and limitation of magnifying endoscopic diagnosis using NBI in the extent of undifferentiated intramucosal gastric adenocarcinoma. *Stomach and Intestine*.

[9] Yagi K, Ajioka Y (2010). *Magnifying Endoscopic Diagnosis in Stomach*.

[10] Lambert R, Rey JF, Sankaranarayanan R (2003). Magnification and chromoscopy with the acetic acid test. *Endoscopy*.

[11] Rey JF, Inoue H, Guelrud M (2005). Magnification endoscopy with acetic acid for Barrett’s esophagus. *Endoscopy*.

[12] Japanese Gastric Cnacer Association (1998). Japanese classification of gastric carcinoma—2nd English edition. *Gastric Cancer*.

[13] Nakayoshi T, Tajiri H, Matsuda K, Kaise M, Ikegami M, Sasaki H (2004). Magnifying endoscopy combined with narrow band imaging system for early gastric cancer: correlation of vascular pattern with histopathology (including video). *Endoscopy*.

[14] Yoshida Y, Kaise M, Kato M (2008). Improvement of diagnosis for early gastric cancer by narrow-band imaging. *Nippon Rinsho*.

[15] Inoue H, Kaga M, Minami H (2008). Endoscopic evaluation of tissue atypia using incorporated-type endocytoscopy for gastric mucosal lesion-NBI magnifying classification and ECA classification. *Nippon Rinsho*.

